# Supplementary material for: Expression of Microtubule-Associated Proteins in Relation to Prognosis and Efficacy of Immunotherapy in Non-Small Cell Lung Cancer
Source: Front Oncol. 2021 Oct 1;11:680402. doi: 10.3389/fonc.2021.680402 (PMC8517487; doi:10.3389/fonc.2021.680402)
Supplement: Supplementary file 1 [file DataSheet_1.zip › immunephenotype/IPS_TCGA-XC-AA0X-01A-32R-A405-07.pdf]

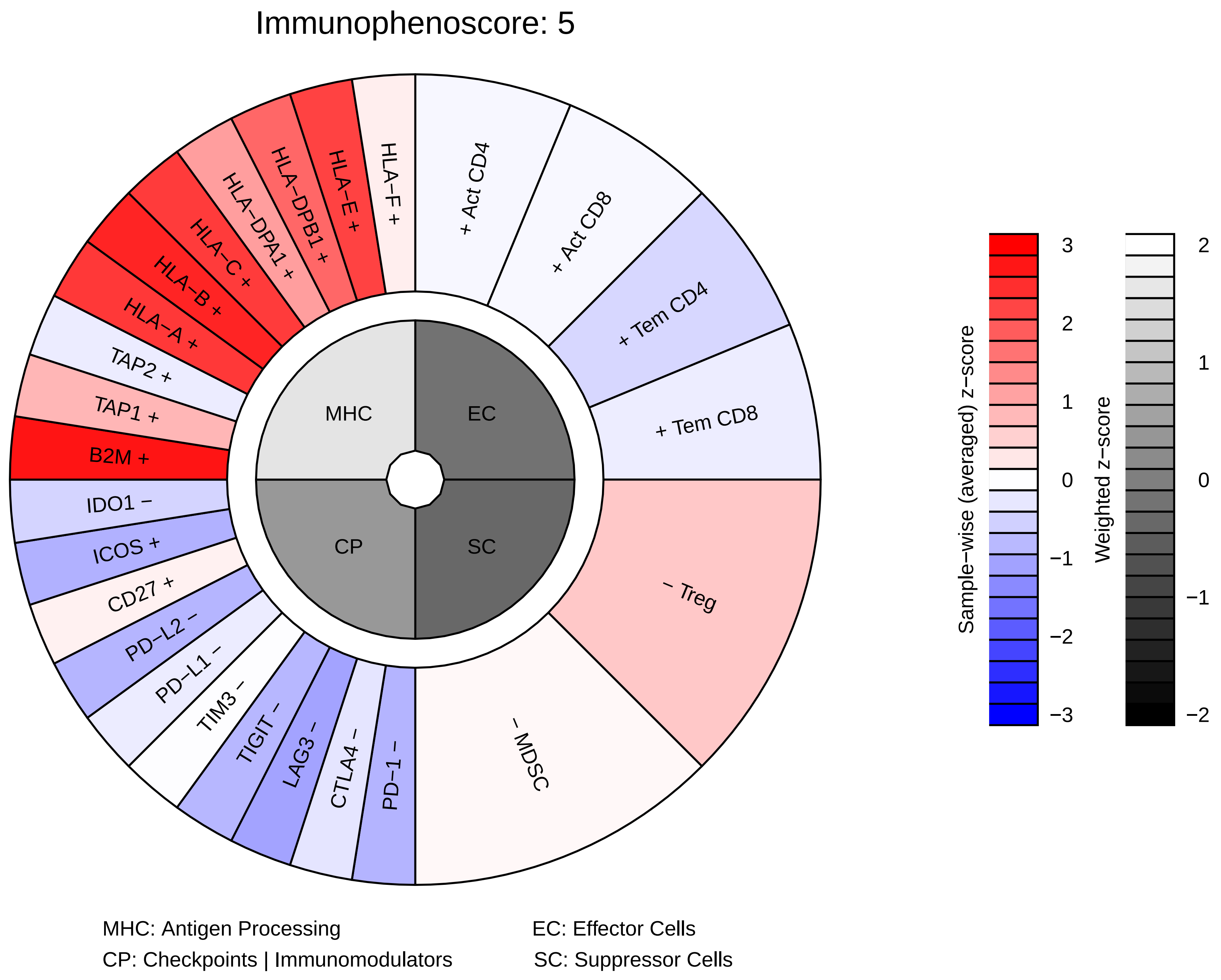
Immunophenoscore: 5
HLA−F +
HLA−E +
HLA−DPB1 +
HLA−DPA1 +
HLA−C +
HLA−B +
HLA−A +
TAP2 +
TAP1 +
B2M +
IDO1 −
ICOS +
CD27 +
PD−L2 −
PD−L1 −
TIM3 −
TIGIT −
LAG3 −
CTLA4 −
PD−1 −
+ Act CD4
+ Act CD8
+ Tem CD4
+ Tem CD8
− Treg
− MDSC
MHC
EC
CP
SC
Sample−wise (averaged) z−score
3
2
1
0
−1
−2
−3
Weighted z−score
2
1
0
−1
−2
MHC: Antigen Processing
CP: Checkpoints | Immunomodulators
EC: Effector Cells
SC: Suppressor Cells
